# Umsetzung eines digitalen Semesters Augenheilkunde während der COVID-19-Pandemie

**DOI:** 10.1007/s00347-020-01316-x

**Published:** 2021-01-15

**Authors:** Andreas Müller, Felix M. Wagner, Juliane Matlach, Marion Zimmermann, Heike Troeber, Katharina A. Ponto, Anita Brill, Franziska Schmidt, Norbert Pfeiffer, Alexander K. Schuster, Verena Prokosch-Willing

**Affiliations:** 1 Augenklinik und Poliklinik der Universitätsmedizin Mainz, Johannes-Gutenberg-Universität Mainz, Langenbeckstr. 1, 55131 Mainz, Deutschland; 2 Zentrum für Qualitätssicherung und -entwicklung, Johannes Gutenberg Universität Mainz, Mainz, Deutschland; 3 Zentrum für Augenheilkunde, Uniklinik Köln, Köln, Deutschland

**Keywords:** Studium, Curriculum, Corona, Digitalisierung, Lehre, Medical school, Curriculum, Teaching, Corona, Digitalization

## Abstract

**Hintergrund:**

Die erste Welle der COVID-19-Pandemie stellte Lehrende und Studierende vor große Herausforderungen, da die studentische Lehre trotz Einschränkung des Präsenzunterrichts stattzufinden hatte. Für Präsenzunterricht und Veranstaltungen mit Patientenkontakt mussten kurzfristig zwischen Mitte März und Beginn des Semesters im April Alternativen gestaltet werden.

**Ziel der Arbeit:**

Beschreibung von Konzept und Umsetzung der studentischen Lehre an der Augenklinik der Universitätsmedizin Mainz in kompletter digitaler Form im Sommersemester 2020.

**Konzeptvorstellung:**

Vorlesung, Untersuchungskurs und Praktikum der Augenheilkunde finden an der Universitätsmedizin Mainz im 5. und 6. Semester im Studiengang Humanmedizin statt. Grundlage der Neukonzeption war der bisherige Kursaufbau. Das umgesetzte Konzept umfasste Vorlesungen als Videopodcasts, Untersuchungsvideos, Online-Untersuchungskonferenzen, leitsymptomorientierte interaktive Patientenfälle, Operationsvideos, Anamnesevideos von Patienten und die Gestaltung eines „Live-Patientenzimmers“, in dem Patientenfälle inklusive Live-Übertragung des Spaltlampenbefundes und der Fundoskopie präsentiert wurden. Die Evaluation durch Studierende zeigte eine sehr gute Annahme des Konzeptes.

**Diskussion:**

Es gelang innerhalb eines Zeitrahmens von 4 Wochen eine vollständige Überarbeitung und Digitalisierung des Kurses Augenheilkunde. Der größte Anteil der Neugestaltung beinhaltete die mediale Produktion von Untersuchungsvideos, interaktiven Patientenfällen und Videopodcasts der Vorlesungen. Diese digitalen Lehrkonzepte können auch in den nächsten Semestern nach Wiederaufnahme des Präsenzunterrichts genutzt werden und die Präsenzlehre in der Augenheilkunde unterstützen.

**Video online:**

Die Online-Version dieses Beitrags (10.1007/s00347-020-01316-x) enthält ein Video.

## Abstract (en)

**Background:**

The first wave of the COVID-19 pandemic posed great challenges for teachers and students, as teaching had to take place despite the restriction of classroom teaching. For attendance lessons and events with patient contact alternatives had to be arranged at short notice between mid-March and the beginning of the semester in mid-April.

**Objective:**

Description of the concept and implementation in the student teaching at the Department of Ophthalmology of the University Medical Center Mainz in complete digital form in spring 2020.

**Presentation of concept:**

Lectures, examination course and practical training in ophthalmology take place in the 5th and 6th semester of the study of human medicine. The basis of the new concept were the former course curricula. Implemented concepts included a complete revision and implementation of lectures as video podcasts, examination videos, online examination conferences, interactive patient cases, narrated videos of surgery, anamnesis videos of patients and the design of the virtual patient room, a live online practice with presentation and examination of patients including transmission of the slit-lamp image to reproduce anterior and posterior segment examination. An evaluation showed a very positive reception of the new concept by students.

**Discussion:**

Within a tight timeframe of 4 weeks a complete revision of the ophthalmology course was achieved. The implementation was time-consuming, with the largest share in the media production of examination videos, interactive patient cases and video podcasts of the lectures. We consider a reduction of classroom teaching for parts of the learning objectives that can be represented by such videos to be possibly useful. An independent digital appropriation of such content may enable a more productive learning environment in face-to-face teaching.

Mit den umfassenden Einschränkungen des öffentlichen Lebens in Deutschland aufgrund der Ausbreitung von COVID-19-Erkrankungen im Frühjahr 2020 konnten auch zahlreiche bisher übliche Lehrformate an den Universitätsklinika zumindest vorübergehend nicht angeboten werden. Daher mussten Alternativen für die bisherigen Lehrkonzepte geschaffen werden.

Die COVID-19-Pandemie hat die Mediziner nicht nur klinisch vor neue Herausforderungen gestellt. Auch die Lehre an den Universitätsklinika musste nach Entscheidung des Bundesgesundheitsministeriums trotz „Lock Down“ und „Social Distancing“ unter schwierigen Bedingungen fortgeführt werden [1]. Es stellte sich die Frage, wie Lehrende die Lernziele ihrer Fächer unter den neuen Auflagen vermitteln könnten. Insbesondere Praktika, Veranstaltungen mit Patientenkontakt und Untersuchungskurse waren zum Semesterstart im April des Jahres 2020 nicht zulässig, obwohl diese an den meisten Universitätsklinika angeboten werden [6]. An der Universitätsmedizin Mainz wurde ein „digitales Semester“ ausgerufen, und die Lehrenden wurden aufgefordert, so viele Kursinhalte wie möglich in einer neuen, digitalen Form anzubieten.

Die Lehre der Universitätsaugenklinik in Mainz wurde auf Basis des vorherigen Curriculums umgestaltet und bot ein ausschließlich digitales Semester mit verschiedenen Lernformaten für die Studierenden an. Dieses Konzept wird im Folgenden vorgestellt, und die Vor- sowie Nachteile werden diskutiert.

## Bisherige Kursstruktur

Die bisherige Lehre der Augenheilkunde fand bis einschließlich des Wintersemesters 2019/20 im 5. und 6. Semester des Humanmedizinstudiums an der Universität Mainz statt.

Im 5. Semester fanden im „Untersuchungskurs der Augenheilkunde“ eine Vorlesungsreihe und ein praktischer Untersuchungskurs mit jeweils 9 Terminen statt. Die Vorlesungen orientierten sich an Funktionen bzw. zugehörigen Untersuchungstechniken und vermittelten propädeutische Grundlagen und theoretische Kenntnisse von ophthalmologischen Untersuchungstechniken anhand beispielhafter Erkrankungen. Diese konnten im Kleingruppenunterricht mit Feedback durch einen ärztlichen Tutor im praktischen Untersuchungskurs aneinander eingeübt werden. Im Kleingruppenunterricht gab es zudem die Gelegenheit, Verständnisprobleme zu Physiologie, Untersuchungen und beispielhaften Erkrankungen zu klären. An digitalen Inhalten standen lediglich die Vorlesungsfolien sowie ein Skript zu Untersuchungstechniken zur Verfügung.

Die Prüfung fand als „objective structured clinical examination“ (OSCE) am Ende des Semesters zu den Inhalten und Untersuchungstechniken von Vorlesung und Kleingruppenunterricht statt.

Im 6. Semester wurden im „Praktikum der Augenheilkunde“ eine Vorlesungsreihe mit 10 Videopodcasts sowie 3 Praktikumstage in der Augenklinik angeboten. Die Vorlesungsreihe orientierte sich an den Organabschnitten des Auges (Lid, Hornhaut, Linse usw.) und den jeweils wichtigsten Erkrankungen dieser. Die Praktikumstage konnten entweder während der vorlesungsfreien Zeit als Blockpraktikum (insgesamt 9 Zeitstunden umfassend) mit eigenständiger Anamnese und Untersuchung von Patienten an der Spaltlampe, Operationshospitation und einem mikrochirurgischen Naht-Wetlab absolviert werden oder während der Vorlesungszeit mit 3 Terminen inklusive eigenständiger Anamnese und Untersuchung von Patienten an der Spaltlampe sowie Sprechstunden- und Operationshospitationen [7]. Das „Mainzer eLearning Augenheilkunde“ stand als digitale Lernressource zur Verfügung mit aktuellen Vorlesungsfolien, beispielhaft beschriebenen Patientenverläufen und Übungsfragen. Links zu externen Lernmaterialien ergänzten das Angebot [8].

Die Prüfung fand im Rahmen einer Präsenz-E-Klausur (Bearbeitung der Klausur am PC in PC-Pool) mit 30 MC-Fragen zu den Inhalten von Vorlesung und Praktikum am Ende des Semesters statt.

## Konzeptvorstellung

Grundsätzliche Ziele der neuen Lehrkonzepte waren die vollständig digitale Umsetzbarkeit, Beibehaltung von Interaktion mit ärztlichen Tutoren und bestmögliche Abbildung von klinisch-praktischen Kompetenzen unter Berücksichtigung des Wegfalls von Präsenzunterricht. Die Universität Mainz vereinheitlichte zudem zeitgleich ihr Learning-Management-System auf eine aktuelle Moodle-Version. Eine Übersicht der Umstrukturierung ist in Tab. 1 abgebildet.

**Table 1:** 

	Bisher	Neukonzeption
*5. Semester*	Vorlesung	Vorlesungsvideopodcast
	Praktischer Untersuchungskurs in Kleingruppen	Untersuchungsvideos
		Online-Tutorium in Kleingruppen
*6. Semester*	Vorlesung	Vorlesungsvideopodcast
	Anamnese und Spaltlampenuntersuchung	Live-Patientenzimmer
		Anamnesevideos
	Operationshospitation	Operationsvideos mit Erklärungen
	Hospitation in der Ambulanz	Interaktive Patientenfälle „red flags“ der Augenheilkunde

### 5./6. Semester: Vorlesungsvideopodcasts

Die Vorlesungsreihen beider Kursteile wurden als wöchentliche Videopodcasts mit Folien zur Präsentation der Inhalte mit erklärender Audiospur konzipiert. Hierbei wurden die bisherigen Vorlesungspräsentationen vollständig überarbeitet. Die Strukturierung anhand von Untersuchungen (5. Semester) bzw. Organstrukturen (6. Semester) wurden beibehalten, wie in Tab. 2 dargestellt.

**Table 2:** 

	Themen
*5. Semester*	Funktionelle Untersuchungen und Refraktion
	Untersuchungen der Lider und Tränenwege
	Untersuchungen der Bindehaut, Hornhaut, Iris und Linse
	Glaukom und Augeninnendruck
	Pupille
	Netzhaut, Sehnerv und Sehbahn
	Motilität und Schielen
	Untersuchungen der Orbita
*6. Semester*	Optik, Refraktion und refraktive Chirurgie
	Lider
	Bindehaut, Tränenwege und Orbita
	Hornhaut
	Sehnerv, Sehbahn und Traumatologie
	Linse
	Glaukom
	Iris, Pupille und Uvea
	Netzhaut
	Motilität

**Figure 1:**
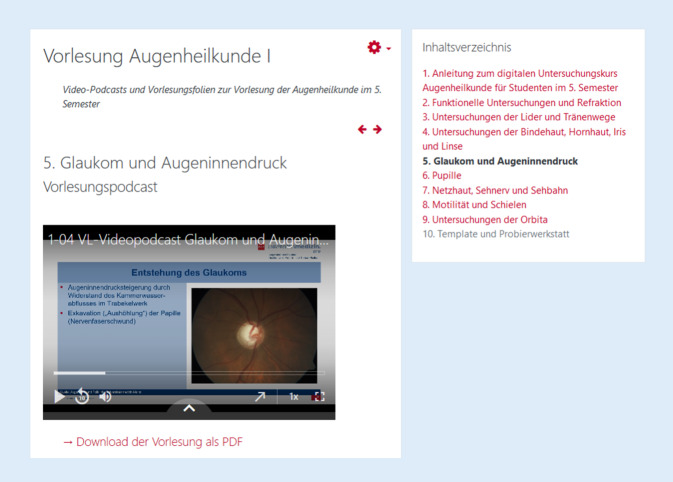

Weiterhin wurde die Gestaltung vereinheitlicht und die Vorlesungsreihen zu wichtigen Inhalten der jeweiligen Themen ausgearbeitet. Die Sprachspur wurde von 2 Dozentinnen (5. Semester) bzw. 1 Dozentin (6. Semester) aufgezeichnet. Die Vorlesungsvideopodcasts wurden wöchentlich als Anker für die jeweils zugeordneten ergänzenden Lernmaterialien auf Moodle veröffentlicht (Abb. 1).

### 5. Semester: Digitaler Untersuchungskurs Augenheilkunde

Als digitale Alternative für den Untersuchungskurs der Augenheilkunde in Kleingruppen wurden Untersuchungsvideos zu essenziellen ophthalmologischen Untersuchungstechniken erstellt: Prüfung des Visus, Messung und Palpation des Augeninnendrucks (Video 1), Konfrontationsperimetrie, Augenmotilitätsprüfung, Prüfung der Pupillomotorik, Untersuchungen bei Verdacht auf Schielen, Brückner-Test, Ektropionieren. Hierbei wurde ein detailliertes Drehbuch von 2 Autoren verfasst und in einem Review-Verfahren nochmals überarbeitet, um eine didaktische Wertigkeit und Nachvollziehbarkeit zu gewährleisten.

Die selbstständige Einübung der Untersuchungen wurde durch ein Online-Tutorium in Kleingruppen unterstützt (Tab. 3). Diese Tutorien wurden mittels Video-Conferencing-Software (z. B. Microsoft Teams [Microsoft Corporation, Redmond, CA, USA]) umgesetzt.

**Table 3:** 

Kursteil	Inhalte
1. Untersuchungsvideos und Vorlesungspodcasts	Theoretische Anleitung mittels Untersuchungsvideo
2. Eigenstudium	Selbstständige Replikation der Untersuchungstechnik an Partnern oder Mitbewohnern
3. Online-Tutorien	Zeigen der individuellen neu erworbenen Untersuchung per Video, Feedback, Besprechung von Hürden und typischen Fehlern

Die Prüfung erfolgte durch die Mentoren in Kleingruppen im Rahmen eines einzelnen Präsenztermins im Juli 2020, bei welchem verbliebene Fragen zu Untersuchungstechniken geklärt wurden und im unmittelbaren Anschluss ein OSCE durchgeführt wurde.

### 6. Semester: Digitales Praktikum der Augenheilkunde

Um den Studierenden Symptome, Zeichen und Krankheitsgeschichten von ophthalmologischen Patienten/-innen näherzubringen, richteten wir 2 Formate ein, in welchen diese zur Sprache kamen. Die Tab. 4 stellt die jeweiligen Inhalte und Abläufe dar.

**Table 4:** 

Kursteil	Inhalte & Ablauf
Live-Patientenzimmer	Ausführliches Anamnesegespräch zu Symptomen und Krankheitsverlauf, welches über Webcam von den Studierenden mitverfolgt werden konnte
	Zweiter Arzt stand Studierenden parallel via Chat zur Verfügung
	Die Studierenden waren aufgefordert, aktiv an der Anamnese teilzunehmen und Fragen via Chat zu stellen, welche vom fragenden Arzt aufgegriffen werden. Klärung von Fragen, welche nicht im Gespräch aufgegriffen wurden, im Chat
	Nach Abschluss des Gesprächs Untersuchung an einer Spaltlampe mit Kameraspion inklusive Fundoskopie mit Live-Übertragung des Bildes per Stream und Live-Besprechung
	Bereitstellung von ergänzenden Informationen durch den nicht untersuchenden Arzt, z. B. in Form von anatomischen Schemata, ebenfalls über den Stream
Anamnesevideos	Freie Anamnese, in welcher die Patientinnen bzw. Patienten v. a. ihre Wahrnehmung der individuellen Erkrankung und Therapien bzw. Operationen berichteten
Operationsvideos	Jeweils ein oder mehrere erklärte ophthalmochirurgische Eingriffe
Interaktive Patientenfälle „red flags“ der Augenheilkunde	Schrittweise Führung der Studierenden durch Patientenvignetten
	Regelmäßige formative Fragen bezüglich zielführender Anamnesefragen, notwendiger Diagnostik oder möglichen therapeutischen Strategien
	Zu jeder Frage jeweils unmittelbar umfangreiches Feedback zu verschiedenen wichtigen Aspekten des Themas

**Figure 2:**
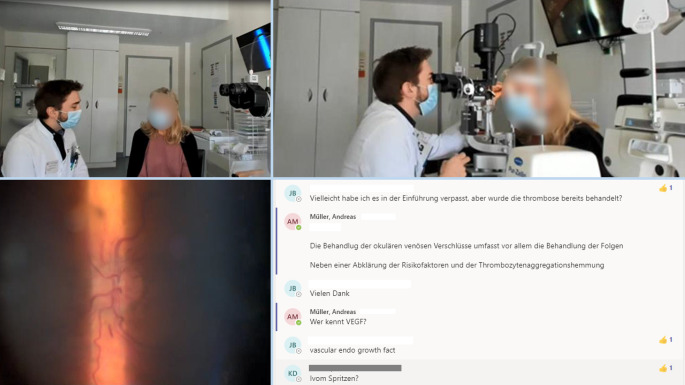

Im „Live-Patientenzimmer“ wurden 2 Patienten mit unterschiedlichen Krankheitsbildern etwa 30 studentischen Teilnehmenden über einen Live-Stream vorgestellt. Das durchführende Team bestand aus mehreren Ärztinnen und Ärzten. Von diesen betreuten jeweils 2 eine Veranstaltung, jeweils einer für Patientenuntersuchung und einer für unmittelbare Betreuung der Studierenden und technische Umsetzung. Mittels Webcam, Mikrofon und Spaltlampenkameraspion wurde die Veranstaltung übertragen. Die Studierenden wurden über einen Live-Chat eingebunden und konnten selbst Fragen stellen (Abb. 2).

Die Patienten waren stationäre Patienten der Augenklinik, welche sich nach einem Aufklärungsgespräch am Vortag über die Veranstaltung und den Rahmen des Datenschutzes schriftlich einverstanden erklären mussten. Die Einwilligung konnte zu jedem Zeitpunkt vor, während oder nach der Lehrveranstaltung zurückgezogen werden. Die Veranstaltungen wurden im Sommersemester 2020 nicht aufgezeichnet und die Studierenden über das Verbot einer Aufzeichnung bei jeder Veranstaltung informiert. Der Datenschutzbeauftragte der Universitätsmedizin Mainz erteilte für das Sommersemester 2020 eine Sondergenehmigung für die Conferencing-Software Microsoft Teams (Microsoft Corporation, Redmond, CA, USA), welche für die Übertragung der Veranstaltung genutzt wurde. Nach Ablauf des Semesters wurde eine datenschutzkonforme Open-Source Alternative für Veranstaltungsformate mit sensiblen Daten geschaffen („Big blue button“, https://bigbluebutton.org/ [BigBlueButton Inc., Open-Source-Software]).

Die „Anamnesevideos“ stellten ein weiteres Format mit echten Patientinnen und Patienten dar. Thematisch wurde jeweils einem Vorlesungsvideopodcast ein Anamnesevideo zugeordnet.

Die Operationsvideos wurden ebenfalls thematisch passend zu den Vorlesungsvideopodcast veröffentlicht.

Als weiteren verpflichtenden Bestandteil des digitalen Unterrichtskonzeptes wurden 7 ophthalmologische Warnsymptome identifiziert:

- Schmerzen nach Trauma
- schmerzhafter Visusverlust beim jungen Menschen
- schmerzhafter Visusverlust beim älteren Menschen
- schmerzloser Visusverlust
- neue Ptose
- Rußregen/Blitze/Vorhangsehen
- rotes Auge

**Figure 3:**
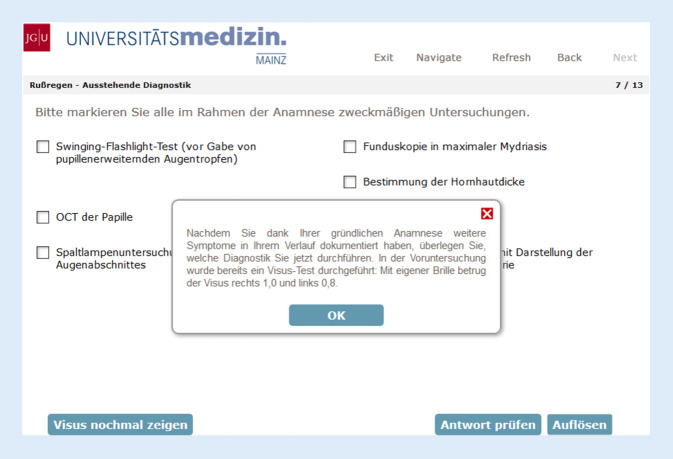

Diese Warnsymptome wurden in Form von interaktiven Patientenfällen („red flags“ der Augenheilkunde) über Moodle zur Verfügung gestellt (Abb. 3). Die Patientenfälle wurden aufgrund ihrer Leitsymptomorientierung den Vorlesungsvideopodcasts nicht spezifisch zugeordnet und parallel veröffentlicht.

Die Klausur fand wie zuvor als E‑Klausur statt, jedoch im Rahmen eines Kohortensystems mit reduzierter gleichzeitiger Teilnehmerzahl, ausreichender Desinfektion und Belüftung der Räumlichkeiten zwischen den einzelnen Prüfungsgruppen.

## Personeller und technischer Aufwand

Es wurden insgesamt ab 24.03.2020 über 6 Wochen 88 Freistellungstage auf 8 Ärztinnen und Ärzte verteilt. Zwei von 8 Ärzten waren durchgehend freigestellt. Die technischen Rahmenbedingungen für einen pünktlichen Start des digitalen Semesters wurden in den ersten 4 Wochen mit 70/88 Freistellungstagen geschaffen. Die letzten beiden Wochen (ab 20.04.) befanden sich bereits in der Vorlesungszeit; 18/88 Freistellungstagen wurden hier genutzt, um die neuen Kursformate durch eingearbeitete Tutoren zu leiten und einen technisch einwandfreien Ablauf zu gewährleisten sowie aufkommende Probleme zu bearbeiten. Semesterbegleitend wurden neben der Patientenversorgung noch einzelne Inhalte finalisiert (z. B. Einsprechen von Vorlesungspodcasts).

Materialien, welche benötigt wurden, sind in Tab. 5 aufgeführt. Diese mussten nur teilweise angeschafft werden, da beispielsweise Kamera und Studiobeleuchtung aus unserer Fotoabteilung entliehen werden konnten.

**Table 5:** 

Zweck	Materialien (^a^bereits vorhanden)	Orientierender Kostenpunkt
Vorlesungspodcasts	Microsoft Office Suite: Powerpoint^a^	Je nach Version
	2 × Sprechermikrofone	2 × 160 €
Dreh-Untersuchungsvideos	Kamera^a^	2000–2500 €
	Richtmikrofon	60 €
	Studiobeleuchtung^a^	200–300 €
	Videoschnittsoftware	Freeware
	Sprechermikrofon	160 €
Interaktive Patientenfälle	„Authoring“-Software	Je nach Software, kostenlose Open-Source-Lösungen verfügbar
	Bildbearbeitungssoftware	Je nach Software, kostenlose Freeware verfügbar
Live-Patientenzimmer	Untersuchungszimmer mit Spaltlampe und Kameraspion^a^	Je nach Ausführung
	Streamingmodul für Kameraspion	2800 €
	Webcam	150–200 €
	Sprechermikrofon	160 €
	Streamingsoftware^a^	Je nach Software, kostenlose Open-Source-Lösungen verfügbar

Abgesehen von einem sicheren Umgang mit einer Office-Suite und ausreichender technischer Versiertheit, um sich in neue, gut dokumentierte Software einzuarbeiten, war kein spezielles „Know-how“ notwendig. Basale HTML-Kenntnisse (Wissen um die grundlegende Struktur von HTML-Code und Websites sind ausreichend) können helfen, die Optik und Funktionalität von browserbasierten eLearning-Inhalten (z. B. interaktive Patientenfälle) zu optimieren.

Für die Konzeption und Umsetzung von Skripten für Untersuchungsvideos und interaktive Patientenfälle sind Erfahrung im Dreh von Lehrvideos und Kenntnisse von Key-feature-Fragenerstellung sinnvoll, welche im Team vorhanden waren.

## Methodik der Evaluation

Es nahmen 64 Studierende im 5. Fachsemester (Untersuchungskurs der Augenheilkunde) sowie 180 Studierende im 6. Fachsemester (Praktikum der Augenheilkunde) an der Evaluation nach Teilnahme an der digitalen Lehre im Juli 2020 teil.

Die Studierenden wurden aufgefordert, nach Abschluss des OSCEs bzw. der Klausur einen Online-Evaluationsbogen auszufüllen, welcher in Zusammenarbeit mit dem Zentrum für Qualitätssicherung und -entwicklung (ZQ) der Johannes-Gutenberg-Universität Mainz gestaltet wurde. Auf diesem wurden zur digitalen Lehre, den einzelnen Bestandteilen des Lehrkonzeptes sowie zur gesamten Veranstaltung Schulnoten vergeben und auf Likert-Skalen Aussagen zur Veranstaltung und der Einschätzung des individuellen Interesses an der Augenheilkunde bewertet. Weiterhin konnten Freitextantworten verfasst werden. Das ZQ wertete die Fragebögen anschließend aus.

Zum Vergleich zu vorigen Semestern wurden vorangegangene allgemeine Evaluationen herangezogen. Diese werden vom Studiendekanat jedes Semesters durchgeführt, wobei Studierende der jeweiligen Kurse zur Teilnahme per Online-Evaluation aufgefordert waren.

## Ergebnisse

### 5. Semester Untersuchungskurs und Vorlesung

Es wurden 64 Evaluationsbögen ausgewertet. Für die jeweiligen Bewertungen konnten zwischen *n* = 57 und *n* = 64 Fragebögen einbezogen werden.

**Figure 4:**
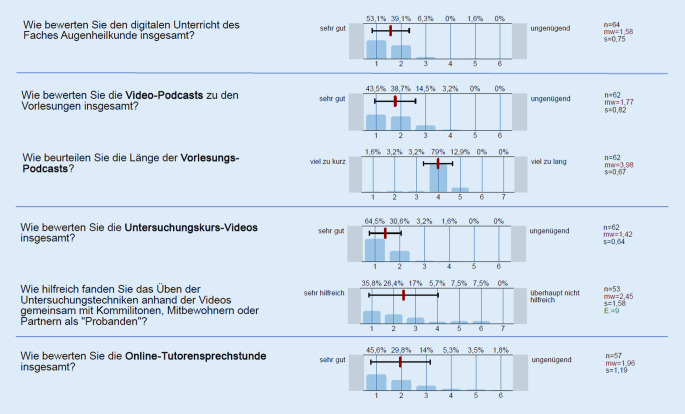

Übergreifend wurde der digitale Unterricht der Augenklinik für den Untersuchungskurs Augenheilkunde im 5. Semester im Mittel mit einer Schulnote von 1,58 ± 0,75 (MW ± SD; 1 = sehr gut, 6 = ungenügend) bewertet (*n* = 64). Die Abb. 4 stellt die Ergebnisse der Evaluation im 5. Fachsemester dar. Die mittlere Länge der Videopodcasts des 5. Semesters betrug 27 min.

### 6. Semester Untersuchungskurs und Vorlesung

**Figure 5:**
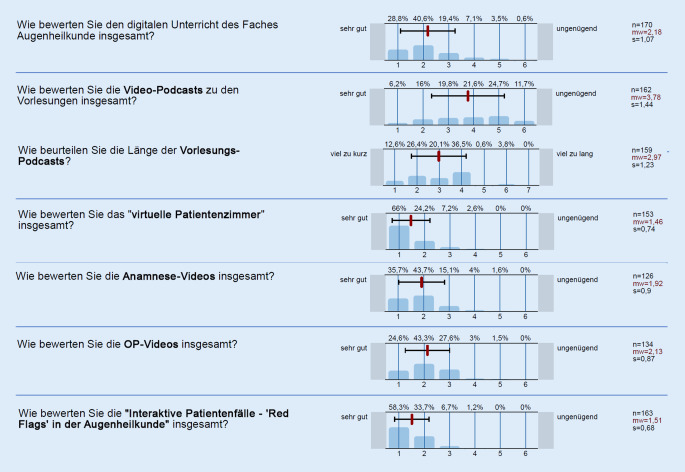

Der digitale Unterricht der Augenklinik für das Praktikum Augenheilkunde im 6. Semester wurde im Mittel mit einer Schulnote von 2,18 ± 1,07 (MW ± SD; 1 = sehr gut, 6 = ungenügend) bewertet (*n* = 170). Die Abb. 5 stellt die Ergebnisse der Evaluation im 6. Fachsemester dar. Die mittlere Länge der Videopodcasts des 6. Semesters betrug 15 min.

### Vergleich zu vorherigen Semestern

Die Ergebnisse der allgemeinen Evaluationen vom Sommersemester 2019 und Wintersemester 2019/20 sind Ergebnissen unserer speziellen Evaluation in Tab. 6 gegenübergestellt.

**Table 6:** 

Kursabschnitt	SS2019	WS2019/20	SS2020
Untersuchungskurs 5. Semester	2,04 ± 1,15 (*n* = 48)	1,75 ± 0,79 (*n* = 56)	1,58 ± 0,75 (*n* = 64)
Praktikum 6. Semester	1,56 ± 0,61 (*n* = 36)	2,14 ± 0,86 (*n* = 59)	2,18 ± 1,07 (*n* = 170)

## Diskussion

Die Erfordernisse einer kompetenzbasierten, modernen Lehre mit Erwerb von praktischen Fertigkeiten inklusive von Kommunikations‑, Handlungs- und Managementkompetenzen stellen der Nationale Kompetenzbasierte Lernzielkatalog Medizin (NKLM) sowie der Lernzielkatalog Augenheilkunde [4, 9]. Auch in Krisenzeiten darf die Zielsetzung der medizinischen Ausbildung nicht in Vergessenheit geraten, da sonst der Erwerb von Schlüsselkompetenzen ganzen Ärztegenerationen vorenthalten wird.

Der Präsenzunterricht in der Medizin ist unersetzlich. Ohne eine persönliche Anleitung und Supervision beim Kompetenzerwerb bleiben die Etablierung einer angemessenen Arzt-Patienten-Kommunikation, angemessenes Verhalten und korrekt vorgenommene Untersuchungstechniken lediglich „try and error“, mit hoher Gefahr von Qualitätseinbußen, insbesondere bei praktischen Tätigkeiten.

Die Rahmenbedingungen der COVID-19-Pandemie verlangten jedoch genau dies und nötigten uns, so viel des Prozesses des Kompetenzerwerbs wie möglich auf mediale, möglichst auch interaktive Elemente auszulagern. Wir achteten hierbei insbesondere auf hohe Qualität (z. B. gute Erkennbarkeit von Untersuchungstechniken in den Videomaterialien) und wertvollen didaktischen Aufbau (z. B. formative Feedbackelemente in den interaktiven Patientenfällen).

Wir erwarteten aufgrund dieses Spannungsfeldes in unserer Evaluation übergreifend trotz intensiver Anstrengungen eine eher negative Entwicklung der Veranstaltungsbewertung. Ausgesprochen erfreulich ist für uns daher insbesondere, dass der Untersuchungskurs Augenheilkunde (5. Semester) ausgezeichnete Rückmeldungen erhielt. Es wurde in Freitextantworten zwar das Fehlen von regelmäßigen Präsenzuntersuchungskursen kritisiert, die Vorbereitung auf Präsenzwiederholungsstunde und OSCE mittels Untersuchungsvideos und Online-Tutorensprechstunde aber deutlich gelobt. Das verbesserte Abschneiden gegenüber den Vorsemestern ist aus unserer Sicht am ehesten durch vollständig aktualisierte und optimierte Vorlesungsinhalte und das neue Angebot der Untersuchungsvideos zu erklären.

Herauszustellen ist aus unserer Sicht außerdem, dass wider unsere Erwartungen das Einüben der Untersuchungstechniken an Kommilitonen, Mitbewohnern oder Partnern im eigenen Haushalt überwiegend als hilfreich für das eigene Lernen angesehen wurde. Dies war lediglich als Notlösung konzipiert, aufgrund des positiven Feedbacks werden wir den Studierenden diesen Schritt im Kompetenzerwerb jedoch auch in kommenden Semestern weiterhin empfehlen.

Das Praktikum der Augenheilkunde konnte sich in seinen Bewertungen in etwa auf dem Niveau des Vorsemesters halten und wurde ebenfalls positiv bewertet. Die Praktikumsmodule (Live-Patientenzimmer, Anamnesevideos, Operationsvideos und interaktive Patientenfälle) wurden sogar durchgehend sehr gut bis gut bewertet.

Das im Verhältnis eher schlechte Abschneiden der Vorlesungspodcasts im 6. Semester sehen wir in der Dauer dieser begründet. Am Beginn des Sommersemesters erhielten die Fakultäten die didaktische Empfehlung, Vorlesungspodcasts auf maximal 15–20 min zu beschränken, da dies etwa der potenziellen Aufmerksamkeitsspanne von Zuhörern entspräche. Für das 5. Semester wurde diese Zeit mit durchschnittlich 27 min Vorlesungspodcastdauer überschritten, in der Evaluation allerdings als ideale Dauer zurückgemeldet.

Die Vorlesungspodcasts des 6. Semesters hielten sich an den Vorschlag mit ca. 15 min. Die Evaluation zeigt, dass die längeren Podcasts deutlich besser angenommen wurden. Wir gehen davon aus, dass Studierende sich das Podcastformat dank Pausierbarkeit individuell eingeteilt haben, weshalb die eigentliche Dauer auch über 20 min hinaus keine Problematik darstellte. Bei Einhaltung einer Maximaldauer von 20 min besteht eine Zwickmühle zwischen umfassender Darstellung eines Themengebietes und dessen didaktischer Aufarbeitung. Aufgrund unserer Erfahrungen ist für einen Vorlesungspodcast daher die Zeitmarke von ca. 30 min als sehr sinnvoll anzusehen.

Auf Basis der positiven Evaluation und Erfahrungen mit den digitalen Unterrichtsinhalten planen wir diese, auch nach dem Ende Corona-bedingter Beschränkungen als ergänzende Angebote zum Präsenzunterricht beizubehalten. Die einzige Ausnahme bilden die Online-Tutorien im 5. Semester, da diese wieder durch einen regelmäßigeren Untersuchungskurs (9 Termine wie im bisherigen Unterrichtskonzept) ersetzt würden. Auch Flipped-classroom-Situationen werden so ermöglicht, in welchen Lerninhalte von den Lernenden zu Hause erarbeitet werden und im Unterricht die Anwendung dieser in den Fokus gerückt wird [2, 3, 5].

Die Erstellung eines umfangreichen digitalen Angebotes erfordert Zeit und Geld. Diese Investition sehen wir jedoch auch unabhängig von der Pandemie als geboten an, da das Ziel von Kompetenzerwerb – wie im NKLM gefordert – unserer Erfahrungen nach durch diese unterstützt werden kann und von den Studierenden positiv angenommen wird. Die Umsetzung wird heutzutage durch eine Vielzahl an einsteigerfreundlichen Softwares unterstützt. So sind auch komplexere mediale und interaktive Inhalte durch die Lehrenden umsetzbar.

## Ausblick

Das laufende Wintersemester 2020/21 wird an der Universitätsmedizin Mainz als hybrides Semester gestaltet. Aufgrund der sehr positiven Bewertungen der digitalen Lehrinhalte behielten wir diese als Ergänzung bei, um eine zeitgemäße digitale Lehre zu ermöglichen, welche sich in ihren Vorzügen mit denen der Präsenzlehre ergänzt. Feedback und Kritik der Studierenden zu den Lehrveranstaltungen werden aufgegriffen und die Lehrangebote entsprechend verbessert. Weiterhin werden die neuen Angebote mit bisherigen inhaltlich besser verknüpft. Außerdem planen wir, die Abdeckung des Lernzielkatalogs Augenheilkunde durch unsere digitalen Angebote zu überprüfen. Nach durchgeführter Optimierung planen wir voraussichtlich für das Sommersemester 2021 erneut eine umfangreiche Evaluation.

## Fazit für die Praxis

- Die Möglichkeiten digitaler Lernmaterialien sind umfangreich, und ein fast ausschließlich digitales Semester konnte durchgeführt werden.
- Besonders der Erwerb praktischer Kompetenzen kann nur teilweise digital realisiert werden.
- Eine Kombination aus digitaler Lehre und ausgewählten Präsenzkursen kann die Vorzüge der Formate kombinieren.

## Supplementary Information


